# EGF released from human placental mesenchymal stem cells improves premature ovarian insufficiency via NRF2/HO-1 activation

**DOI:** 10.18632/aging.102794

**Published:** 2020-02-10

**Authors:** Chenyue Ding, Qinyan Zou, Yifei Wu, Jiafeng Lu, Chunfeng Qian, Hong Li, Boxian Huang

**Affiliations:** 1 Center of Reproduction and Genetics, Affiliated Suzhou Hospital of Nanjing Medical University, Suzhou Municipal Hospital, Suzhou 215002, China; 2 State Key Laboratory of Reproductive Medicine, Nanjing Medical University, Jiangsu 210029, China

**Keywords:** human placental mesenchymal stem cells, premature ovarian insufficiency, epidermal growth factor, oxidative stress, NRF2/HO-1

## Abstract

Human placental mesenchymal stem cells (hPMSCs) have the ability to release cytokines and to differentiate into the three germ layers. To date, the relevance of hPMSCs for the treatment of premature ovarian insufficiency (POI) disease through the regulation of oxidative stress is still unclear. Therefore, to evaluate the therapeutic efficiency and investigate the mechanism of hPMSCs, we generated a mouse model of POI and collected human ovarian granule cells (hGCs) from patients with POI. hPMSCs displayed therapeutic effects on POI ovarian function, including recovered follicular numbers and increased expression of oocyte markers. Furthermore, secretion of the cytokine EGF (epidermal growth factor) was higher from hPMSCs than it was from other cells. FACS and Western blot analyses showed that EGF elevated the proliferation and reduced the apoptosis in hGCs. hPMSCs and EGF inhibited oxidative stress levels. Protein assays demonstrated that EGF suppressed oxidative stress by dose-dependently upregulating the expression of the NRF2/HO-1 pathway, and it inhibited the apoptosis by regulating the PTEN/PI3K/AKT pathway. These findings provide an experimental foundation for hPMSCs in improving ovarian function through the secretion of EGF. The mechanism of action of EGF is related to protection from oxidative stress by activation of the NRF2/HO-1.

## INTRODUCTION

Current epidemiological data show that the ovary plays a vital role in regulating metabolic and physiological functions in women [1]. Thus, there is no surprise that abnormalities in ovarian function can contribute to increasing the risk of disease susceptibility. Premature aging is the progressive loss of tissue and organ function. Premature ovarian insufficiency (POI) is characterized by a significant decrease in the oocyte reserve, ovarian follicle pool and telomere length, which leads to reproductive senescence that occurs before death in most species [2]. DNA damage can accumulate over time, but in resting cells, such as human granule cells (hGCs) or primordial follicles, there is the inability to eliminate faulty cells during replication, which results in premature ovarian failure [3].

Human placental mesenchymal stem cells (hPMSCs) have been proven to have therapeutic potential in preclinical models [4]. Some studies have applied mesenchymal stem cells to the repair of damaged ovaries in rats or of POI hGCs; types of mesenchymal stem cells used in these studies include human umbilical cord mesenchymal stem cells, human amniotic mesenchymal stem cells and human amniotic fluid stem cells [5, 6]. When lesions occur, MSCs can alleviate tissue damage by stimulating the recruitment and proliferation of endogenous stem cells, inhibiting fibrotic remodeling and apoptosis, promoting antiapoptotic activity, and reducing the immune response [7]. The characteristics of poor immunogenicity and stable proliferation make hPMSCs a new source of stem cells that are suitable for cell therapy. Moreover, their non injurious property also holds substantial prospects self-repair of cells and regeneration. MSCs participate in the tissue repair process through the release of cytokines or related proteins directly or indirectly [8]. The cytokine epidermal growth factor (EGF) not only is essential for oocyte maturation, oogenesis, and fertility but can also induce cumulus cell expansion in vitro [9]. A previous study also showed that EGF-like genes in cumulus cells are considered essential for maintaining an autocrine signaling loop and the progression of oocyte maturation [10]. In addition, EGF family members promote growth and differentiation of recruited primordial follicles in late folliculogenesis [11].

In humans, it has been proven that the most likely contribution to premature ovarian failure is oxidative stress, which is induced by reactive oxygen species (ROS); however, the molecular mechanism that decreases the quality and quantity of oocytes remains unclear [12]. It has been demonstrated that oxidative stress is associated with various age-related pathological diseases. A previous study has shown that POI was implicated in decreased antioxidant levels and increased oxidative stress (OS) in the cumulus cells, oocytes and ovaries in POI disease, and higher OS levels were related to worse outcomes [13]. DNA and proteins may be damaged by oxidative stress generated under these stressful conditions, and as a result, the cellular processes are disrupted [14]. Nuclear factor erythroid 2-related factor 2 (NRF2), a transcription factor, is responsible for balancing cellular redox as well as controlling phase II detoxification responses and antioxidant and detoxification enzymes in mammals [15]. The NRF2 signaling system has the capacity to protect against oxidative stress and toxicants and is thus considered to be important for cellular defense and survival [16]. Disruption of NRF2 signaling is related to susceptibility to oxidative damage in humans and model organisms [17]. In addition, as a representative NRF2-target gene, heme oxygenase-1 (HO-1), with its ability to reverse oxidative damage and stress [18], has also been shown to play critical roles in antioxidant defense and various pathophysiologic processes in an age-related manner [19]. Therefore, the relationship between aging and HO-1 regulation has become a popular area of research in recent decades.

Nevertheless, little is known regarding the association between hPMSCs and POI at the oxidative stress level. Therefore, our study aims to identify whether hPMSCs are capable of recovering POI mediated by the NRF2/HO1 pathway.

## RESULTS

### hPMSCs induced oogenesis in a POI mouse model

**Figure 1:**
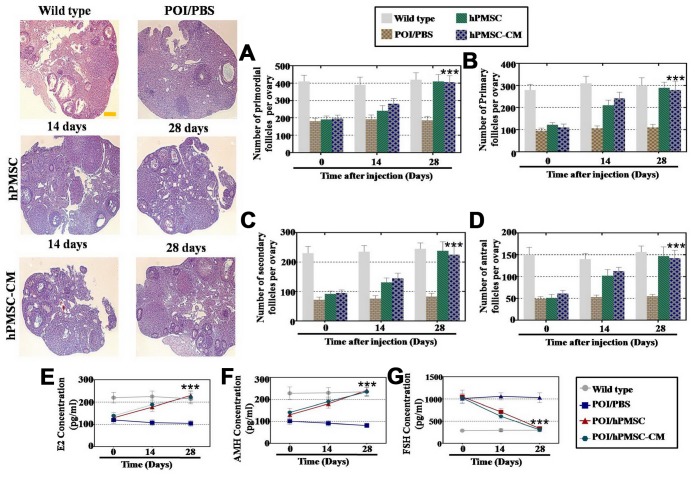
**hPMSCs improved the function of a POI mouse model.** (**A**) The number of primordial follicles recovered to normal levels four weeks after hPMSC transplantation. (**B**) hPMSC transplantation restored the primary follicle numbers. (**C**) hPMSC elevated the number of secondary follicles to the WT group level (**D**) hPMSC transplantation elevated the number of antral follicles to the WT group level. (**E**) ELISA results indicated that hPMSC transplantation increased the levels of E2. (**F**) hPMSC transplantation improved the levels of AMH. (**G**) hPMSC transplantation decreased the levels of FSH to the WT group level. All experiments were carried out three times; error bars indicate the SD; *** *p* < 0.001 (compared with the POI group). POI = premature ovarian insufficiency, hPMSC = human placental mesenchymal stem cell.

First, we employed FACS to evaluate the therapeutic effect of hPMSCs in a POI mouse model. Our results indicated high expression of cell surface markers CD29, CD73 and CD90 and minimal expression of CD105 and CD34 in hPMSCs (Supplementary Figure 1A). hPMSCs with the capacity to differentiate into adipocytes, chondroblasts and osteoblasts have been demonstrated to be multipotent mesenchymal stem cells (Supplementary Figure 1B). Ovarian tissues were HE stained, and the results revealed that the follicle number was significantly restored by treatment with hPMSCs and hPMSCs-CM at the fourth week (Figure 1A–1D). The level of hormones in the plasma was measured after hPMSC transplantation. From one to four weeks, AMH and E2 gradually recovered to normal levels following treatment with hPMSCs (Figure 1E and 1G). Compared to the control group, FSH was significantly reduced and returned to normal levels (Figure 1F). Thus, hPMSCs exhibited a capacity for restoring the functioning ovary in POI mice.

### hPMSCs improved the expression levels of markers in POI-hGCs

**Figure 2:**
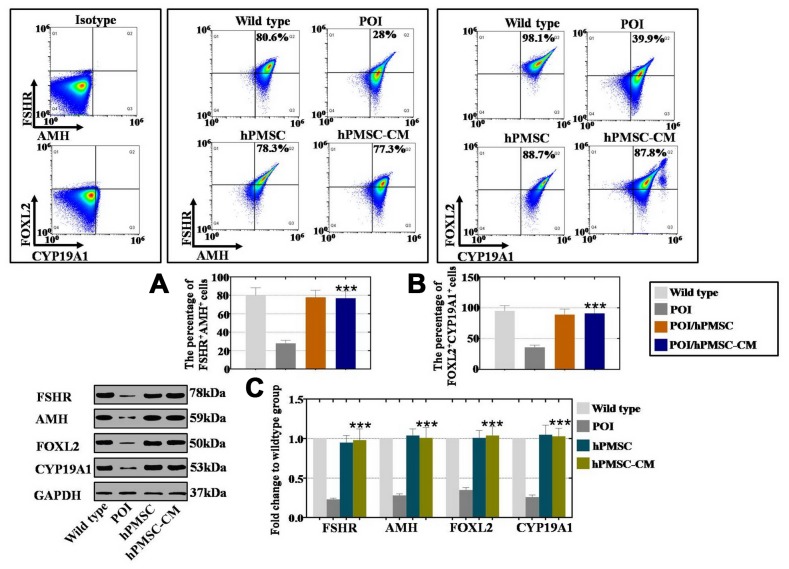
**hPMSCs upregulated the marker expression level of hGCs with POI.** (**A**) FACS results indicated that hPMSCs increased the number of FSHR^+^AMH^+^ positive hGCs. (**B**) FACS results indicated that hPMSCs increased the number of CYP19A1^+^FOXL2^+^ positive hGCs. (**C**) Western blot results showed that hPMSCs increased the protein levels of FSHR, AMH, CYP19A1 and FOXL2 in POI hGCs to the normal level. All experiments were carried out three times; error bars indicate the SD; *** *p* < 0.001 (compared with the POI group). POI = premature ovarian insufficiency, hPMSC = human placental mesenchymal stem cell, hGCs = human ovarian granule cells.

To explore the treatment effects of hPMSCs on POI patients, our researchers collected hGCs from normal and POI patients from our reproductive medicine center, and changes were detected in the expression of markers after coculture with hPMSCs, as previously reported [6]. The hGC markers AMH (follicular growth), FSHR (follicular maturation), FOXL2 (follicular activation) and CYP19A1 (ovary formation) were used to evaluate the effects via FACS and Western blot analysis. As shown in Figure 2A, the results of FACS analysis showed that a larger increase was observed in the FSHR^+^AMH^+^ cell number of the hPMSCs and hPMSCs-CM groups (78% and 77%, respectively) than was observed in the POI group (28%). In Figure 2B, the increase in the number of FOXL2^+^CYP19A1^+^ cells (88% and 87%, respectively) was greater in the hPMSCs and hPMSC-CM groups than it was in the POI group (39%). Western blot analysis indicated similar results: hPMSCs and hPMSC-CM treatment increased the expression of FSHR, AMH, FOXL2 and CYP19A1 to levels similar to that of the WT group, as shown in Figure 2C. In conclusion, hPMSCs restored the marker expression of hGCs.

### EGF derived from hPMSCs was highly expressed in CM

**Figure 3:**
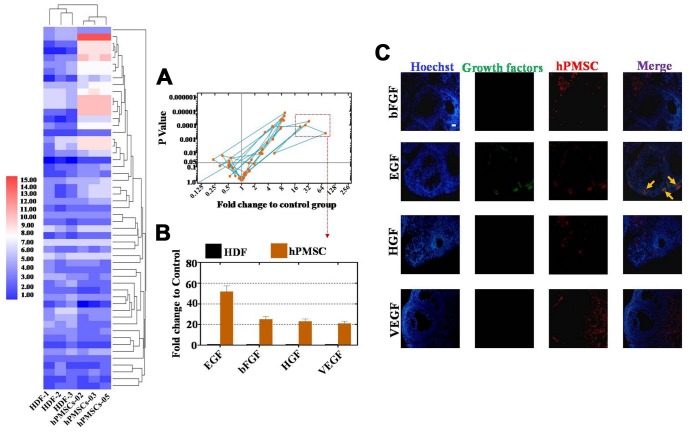
**EGF derived from hPMSCs was observed at higher levels than other growth factors.** (**A**) Antibody microarray analysis of growth factor secretion from the hPMSC and control groups (293T cell line). Four growth factors were selected in accordance with standard criteria: the fold change was greater than or equal to sixteen, and there was statistical significance (*p*< 0.01). (**B**) EGF derived from hPMSCs was secreted at higher levels than other growth factors. (**C**) EGF was highly expressed in the POI mouse model after hPMSC transplantation. All experiments were carried out three times. hPMSC = human placental mesenchymal stem cell.

To further elaborate the mechanism of the hPMSC transplantation effects on a POI mouse model, we collected the supernatant of three hPMS cell lines from individual donors (including 1 male and 2 female donors) and an HDF cell line as a control group. Expression profiles were assessed with a cytokine antibody array (growth factors = 53). Our results elucidated that hPMSCs secreted more growth factors than the control group (Figure 3A). hPMSCs secreted twenty-two growth factors, which had substantially higher secretion levels than what was observed from the control group (*p* < 0.05) in Figure 3A. We selected four growth factors, EGF, bFGF, HGF, and VEGF, with fold changes greater than or equal to ten that also exhibited a statistical significance when comparing among groups (*p* < 0.01) (Figure 3B). Of all the growth factors, EGF was secreted at the highest level (Figure 3B). In addition, four weeks after hPMSCs were transplanted into mice, immunofluorescence assays were performed, and the data indicated that among the four growth factors, only EGF was expressed in the ovary (Figure 3C). Collectively, these results showed that EGF derived from hPMSCs may be responsible for ameliorating ovarian function.

### EGF derived from hPMSCs led to improved proliferation and inhibition of apoptosis in hGCs

**Figure 4:**
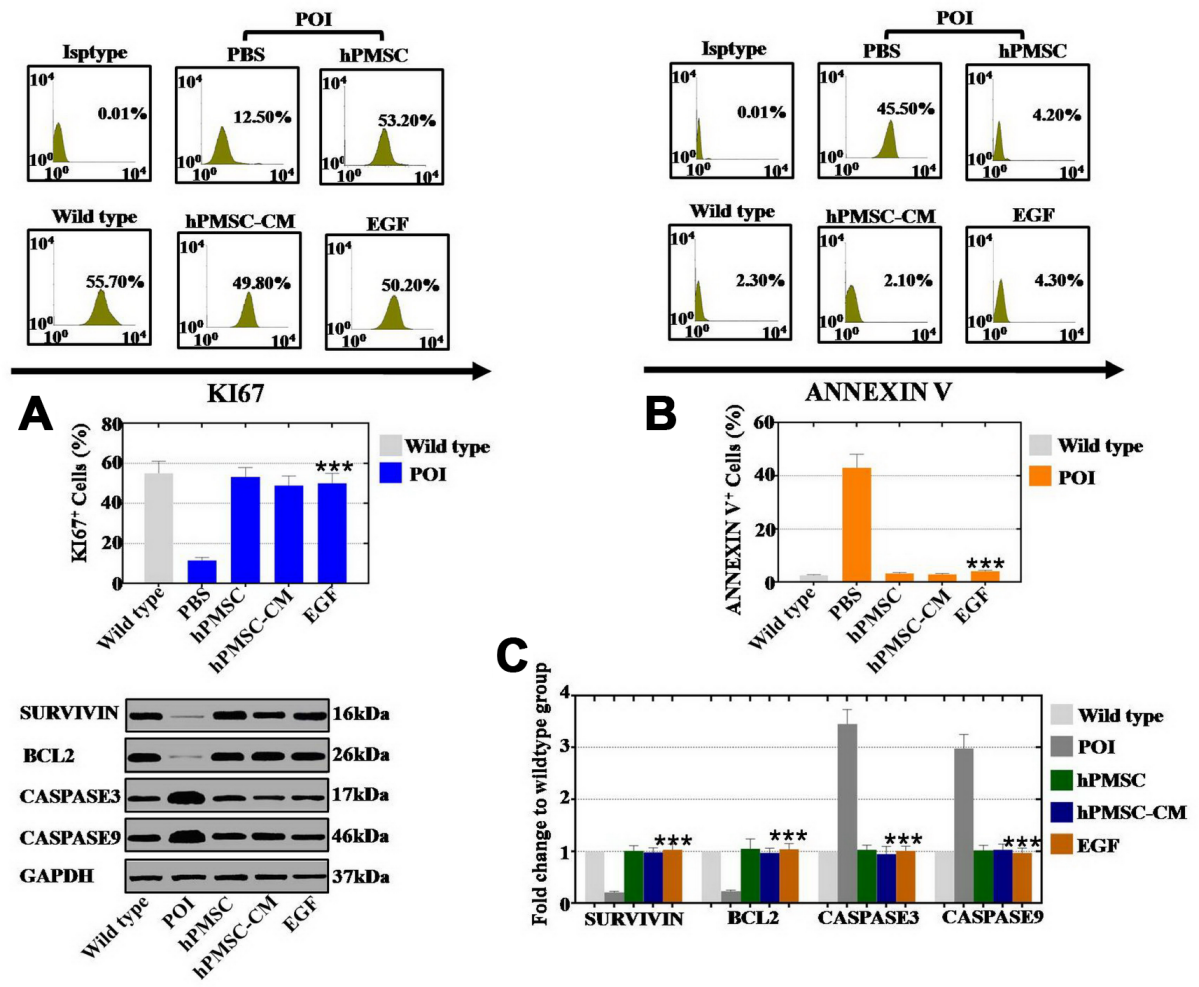
**EGF derived from hPMSCs improved the proliferation rate and inhibited the apoptosis rate in POI hGCs.** (**A**) FACS results indicated that hPMSCs, hPMSC-CM or EGF treatment improved the proliferation rate (Ki67) in POI hGCs. (**B**) FACS results indicated that hPMSCs, hPMSC-CM or EGF treatment inhibited the rate of apoptosis (Annexin V) in POI hGCs. (**C**) Western blot results demonstrated that hPMSCs, hPMSC-CM or EGF treatment increased the expression of apoptosis resistance genes (Bcl2 and Survivin) and reduced the expression levels of apoptosis genes (Caspase 3 and Caspase 9). All of the experiments were carried out three times; the error bars indicate the SD; *** *p* < 0.001 (compared with the POI group). POI = premature ovarian insufficiency.

We further cocultured hGCs with hPMSCs, hPMSC-CM and EGF for 7 days to investigate the role of hPMSCs in cell proliferation and apoptosis. We employed FACS analysis to quantitatively assess cell viability. The proliferation rates of hGCs in the hPMSC group, in the hPMSC-CM group and in the EGF group was increased to 53%, 48% and 50%, respectively, which were higher than that of the POI group (11%) (Figure 4A). As shown in Figure 4B, the rates of apoptosis in the hPMSC group, hPMSC-CM group and EGF group were clearly decreased to 3.2%, 2.9% and 4.1%, respectively, which was lower than that of the POI group (43%). We also used a protein level assay to evaluate the expression of apoptosis- related genes (CASPASE 3 and CASPASE 9) and apoptosis resistance-related genes (BCL2 and SURVIVIN). After separate cocultures of hPMSCs, hPMSCs-CM and EGF with POI-hGCs, the expression levels of BCL2 (105%, 97% and 104%, respectively) and SURVIVIN (101%, 98% and 103%, respectively) were improved compared to those in the WT group (23% and 21% for BCL2 and SURVIVIN, respectively) (Figure 4C). Furthermore, compared to the WT group, hPMSCs, hPMSCs-CM and EGF had stronger inhibitory effects on the expression levels of CASPASE 3 (103%, 95% and 101%, respectively) and CASPASE 9 (102%, 103% and 97%, respectively) than the POI group (345% and 298% for CASPASE 3 and CASPASE 9, respectively) (Figure 4C). In summary, hPMSCs could elevate the rate of proliferation and suppress the apoptotic effects of hGCs, and EGF exhibited similar effects.

### hPMSCs inhibited ROS in hGCs by secreting the cytokine EGF

**Figure 5:**
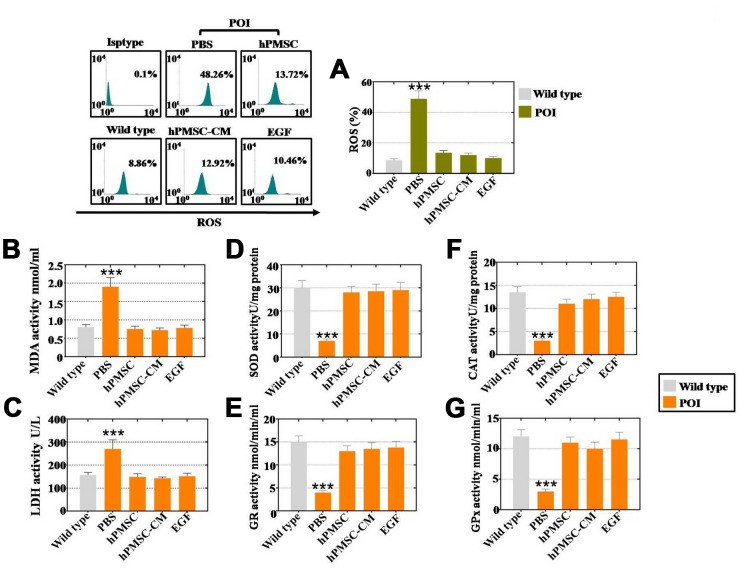
**EGF derived from hPMSCs suppressed ROS in POI hGCs.** (**A**) hPMSC, hPMSC-CM or EGF treatment suppressed ROS in vitro to normal levels (POI hGCs). (**B**) ELISA results revealed that hPMSCs, hPMSC-CM or EGF treatment inhibited MDA expression to the WT group level. (**C**) hPMSC, hPMSC-CM or EGF treatment suppressed the level of SOD expression in vitro. (**D**) ELISA results indicated that hPMSCs, hPMSC-CM or EGF treatment elevated CAT expression in POI hGCs. (**E**) hPMSC, hPMSC-CM or EGF treatment increased LDH expression to the WT group level in vitro. (**F**) ELISA results demonstrated that hPMSCs, hPMSC-CM or EGF improved the level of GR in vitro. (**G**) hPMSC, hPMSC-CM or EGF treatment increased GPx expression to normal levels in POI hGCs. All of the experiments were carried out three times. The error bars indicate the SD; *** *p* < 0.001 (compared with the POI group). POI = premature ovarian insufficiency.

Our subsequently explored whether hPMSCs improved the vitality of hGCs by inhibiting ROS. As shown in Figure 5A. Coculture with hPMSCs, hPMSCs-CM and EGF repressed ROS to 13%, 12% and 10%, respectively, as detected by FACS. The activity of oxidative and antioxidative enzymes was assessed as well. The results of the ELISAs indicated that the expression of oxidative enzymes (MDA and LDH) was reduced nearly to the levels observed in the WT group by hPMSC, hPMSC-CM and EGF treatment, as shown in Figure 5B and 5C. Moreover, hPMSCs, hPMSCs-CM and EGF increased the expression level of antioxidative enzymes (SOD, GR, CAT and GPx) to levels that are normal, as compared to the WT group in Figure 5D–5G. Therefore, hPMSCs can reduce ROS levels, resist oxidative enzyme activity and improve the levels of antioxidant enzymes by secreting EGF.

### EGF derived from hPMSCs repressed ROS by upregulating the NRF2/HO-1 pathway in vitro

**Figure 6:**
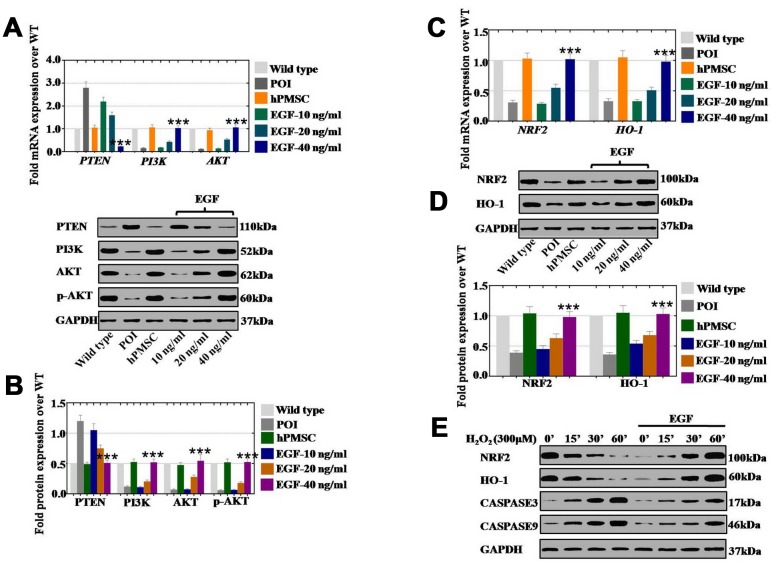
**EGF derived from hPMSCs suppressed ROS by upregulating the NRF2/HO-1 pathway in vitro.** (**A**) qPCR analysis of the mRNA expression levels of PI3K, AKT and PTEN in POI hGCs after hPMSC or EGF (with 10 ng/ml, 20 ng/ml, and 40 ng/ml) treatment. (**B**) Western blot analysis of the protein levels of PI3K, AKT and PTEN in POI hGCs after hPMSC and EGF (with 10 ng/ml, 20 ng/ml, and 40 ng/ml) treatment. (**C**) qPCR analysis of the mRNA levels of NRF2 and HO-1 in POI hGCs after hPMSC or EGF (with 10 ng/ml, 20 ng/ml, and 40 ng/ml) treatment. (**D**) Western blot analysis of the protein levels of NRF2 and HO-1 in POI hGCs after hPMSC or EGF (with 10 ng/ml, 20 ng/ml, and 40 ng/ml) treatment. (**E**) Western blot analysis of the protein levels of NRF2, HO-1, CASPASE 3 and CASPASE 9 in H_2_O_2_-treated hGCs after EGF (with different concentrations) coculture. All experiments were carried out three times. The error bars indicate the SD; *** *p* < 0.001 (compared with the POI group). POI = premature ovarian insufficiency. The qPCR primer sequences used are listed in Supplementary Table 1.

To reveal how hPMSCs positively impacted POI disease, hPMSCs and EGF at three concentrations (10 ng/ml, 20 ng/ml, and 40 ng/ml) were cocultured with POI hGCs for 7 days. The results of qPCR assays showed that hPMSCs decreased the expression of PTEN (105%) and elevated the expression levels of PI3K (106%) and AKT (94%) to levels similar to those of the wild-type group (Figure 6A). In addition, the expression of PTEN was gradually inhibited to 95%, 39% and 23% in a dose-dependent manner (EGF at 10, 20, and 40 ng/ml, respectively) (Figure 6A). The expression of PI3K and AKT was significantly elevated to 18%, 43%, and 103% and to 14%, 53%, and 106%, respectively, in a dose-dependent manner (EGF at 10, 20, and 40 ng/ml, respectively) (Figure 6A). Moreover, similar results were revealed at the protein level, as shown in Figure 6B. The NRF2/HO-1 pathway was detected after treatment with hPMSCs or EGF in POI hGCs, and we monitored NRF2 and HO-1 mRNA and protein levels by qPCR and Western blot. We observed a 103% and 105% increase in NRF2 and HO-1 mRNA levels, respectively (Figure 6C), and increases of 104% and 105% in NRF2 and HO-1 protein levels, respectively, following treatment with hPMSCs, as shown in Figure 6D. Furthermore, the expression of NRF2 and HO-1 was tested after coculture with different EGF concentrations by qPCR assays and Western blotting. The mRNA expression of NRF2 and HO-1 was significantly elevated to 29%, 55%, and 102% and to 33%, 51%, and 98%, respectively, following the increases in EGF concentration (10, 20, and 40 ng/ml, respectively) relative to the control group (Figure 6C). The protein expression levels of NRF2 and HO-1 were substantially increased to 45%, 63%, and 98% and to 54%, 68%, and 103%, respectively, in a concentration-dependent manner (10, 20, and 40 ng/ml, respectively) (Figure 6D). We further performed Western blots in wild-type hGCs to determine whether EGF could resist cell damage induced by H_2_O_2_. H_2_O_2_ exhibited an inhibitory effect on the expression of NRF2/HO-1 and apoptosis genes (Caspase 3 and Caspase 9) in a time-dependent manner (treatments for 0’, 15’, 30’, and 60’). However, EGF (40 ng/ml) clearly returned NRF2/HO-1 and apoptosis genes to regular levels in a time-dependent manner (Figure 6E).

### EGF derived from hPMSCs repressed ROS by upregulating the NRF2/H O-1 pathway in vivo

**Figure 7:**
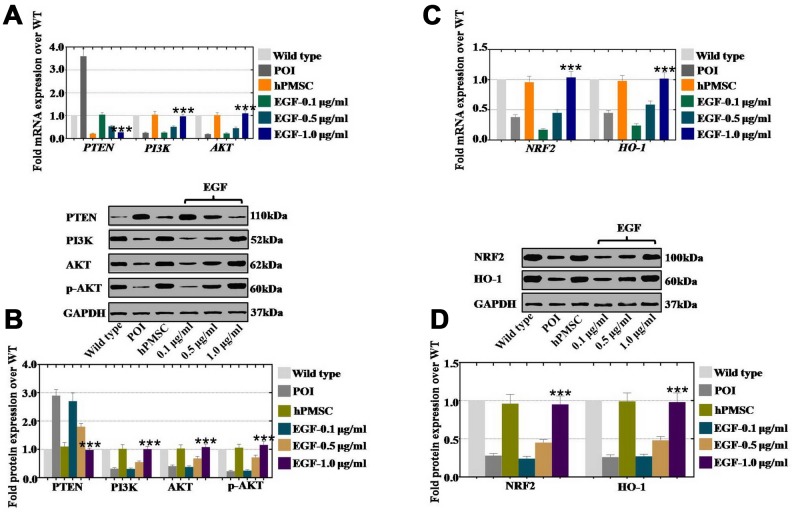
**EGF derived from hPMSCs suppressed ROS by upregulating the NRF2/HO-1 pathway in a POI mouse model.** (**A**) qPCR analysis of the mRNA expression levels of PI3K, AKT and PTEN in POI hGCs after in vivo treatment with hPMSC or EGF (with 0.1 μg/ml, 0.5 μg/ml, and 1.0 μg/ml). (**B**) Western blot analysis of the protein expression levels of PI3K, AKT and PTEN in POI hGCs after hPMSC or EGF (with 0.1 μg/ml, 0.5 μg/ml, 1.0 μg/ml) treatment. (**C**) qPCR analysis of the mRNA levels of NRF2 and HO-1 in POI hGCs after hPMSC and EGF (with 0.1 μg/ml, 0.5 μg/ml, and 1.0 μg/ml) treatment. (**D**) Western blot analysis of the protein levels of NRF2 and HO-1 in POI hGCs after hPMSC and EGF (with 0.1 μg/ml, 0.5 μg/ml, and 1.0 μg/ml) treatment. All experiments were carried out three times. The error bars indicate the SD; *** *p* < 0.001 (compared with the POI group). POI = premature ovarian insufficiency. The qPCR primer sequences used are listed in Supplementary Table 1.

hPMSCs and EGF at three concentrations (0.1 μg/ml, 0.5 μg/ml, and 1.0 μg/ml) were injected into POI ovaries for one month to investigate how hPMSCs improved ovarian function. Compared to the wild-type group, hPMSCs decreased the expression of PTEN (21%) and markedly improved the expression of PI3K (104%) and AKT (102%) compared to the POI group (360% in PTEN, 25% in PI3K, 19% in AKT) (Figure 7A). In addition, the expression of PTEN also displayed a significant inhibition (104%, 53%, and 27%), and PI3K and AKT were significantly elevated to 26%, 51%, 97% and to 22%, 45% 110%, respectively, following the EGF concentration increase (0.1 μg/ml, 0.5 μg/ml, and 1.0 μg/ml, respectively) (Figure 7A). Moreover, the protein results were the same as those observed by qPCR. The expression of PTEN, PI3K and AKT was returned to the WT group level in the hPMSC and EGF treatment groups. There was a dose-dependent effect in the EGF group, as shown in Figure 7B. Furthermore, the relative regulatory genes (NRF2 and HO-1) were also assessed via qPCR and Western blot. At the mRNA level, hPMSCs increased the expression of NRF2 (96%) and HO-1 (98%) more dramatically than what was observed in the POI group (38% and 45%), as compared to the WT group (Figure 7C). Moreover, the expression levels of NRF2 and HO-1 were significantly increased to 17%, 45%, 104% and to 24%, 59%, 102%, respectively, in the different EGF concentration treatment groups (0.1 μg/ml, 0.5 μg/ml, and 1.0 μg/ml, respectively) (Figure 7C). In addition, hPMSCs increased the protein expression of NRF2 (96%) and HO-1 (99%) to higher levels than were observed in the POI group (28% and 26%, respectively) (Figure 7D). The expression of NRF2 and HO-1 was significantly elevated to 24%, 45%, and 95% and to 27%, 48%, and 98%, respectively, in a concentration-dependent manner (EGF with 0.1 μg/ml, 0.5 μg/ml, and 1.0 μg/ml, respectively) in Figure 7D.

### EGF derived from hPMSCs had a minimal impact on the ROS level in hGCs after NRF2 knockdown

**Figure 8:**
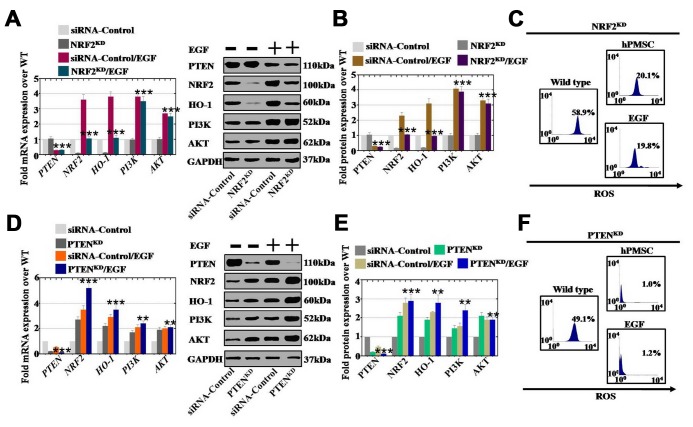
**EGF affected ROS levels by regulating the NRF2/HO-1 signaling pathway but not the PI3K/AKT signaling pathway.** (**A**) qPCR analysis of the expression levels of PTEN, NRF2, HO-1, PI3K and AKT in hGCs-NRF2^KD^ after treatment with EGF. (**B**) Western blot analysis of the expression levels of PTEN, NRF2, HO-1, PI3K and AKT in hGCs-NRF2^KD^ after treatment with EGF. (**C**) ROS levels were measured in hGCs-NRF2^KD^ after treatment with hPMSCs and EGF. (**D**) qPCR analysis of the expression levels of PTEN, NRF2, HO-1, PI3K and AKT in hGCs-PTEN^KD^ after treatment with EGF. (**E**) Western blot analysis of the expression levels of PTEN, NRF2, HO-1, PI3K and AKT in hGCs-PTEN^KD^ after treatment with EGF. (**F**) ROS levels were measured in hGCs-PTEN^KD^ after treatment with hPMSCs and EGF. All experiments were carried out three times. The error bars indicate the SD; **, *p* < 0.01; *** *p* < 0.001 (compared with the NRF2 or PTEN knockdown group, respectively). The qPCR primer sequences used are listed in Supplementary Table 1.

To determine whether EGF drives PTEN and NRF2 expression in an appropriate context, we performed an siRNA assay to establish human ovarian granular cell lines with NRF2 and PTEN knocked down. Except for the expression of PI3K and AKT, NRF2^KD^ inhibited the expression of NRF2 and HO-1 compared to that of the siRNA control group, as assessed by qPCR and Western blot assays (Figure 8A and 8B). After using EGF to treat hGCs-NRF2^KD^, the expression of NRF2 and HO-1 was found to be slightly elevated at both mRNA and protein levels, while NRF2^KD^ did not influence the mRNA and protein levels of PI3K and AKT with or without EGF treatment (Figure 8A and 8B). As shown in Figure 8C, after coculture with hGCs-NRF2^KD^, hPMSCs and EGF inhibited the level of ROS (20% and 19%, respectively) compared to the WT group (58%), as shown by FACS analysis. To confirm that EGF derived from hPMSCs repressed ROS through the PTEN signaling pathway, we established a human ovarian granular cell line with PTEN knocked down. PTEN^KD^ increased the mRNA expression of NRF2 (270%), HO-1 (220%), PI3K (170%) and AKT (190%) compared to the siRNA control group (Figure 8D). As shown in Figure 8D, after EGF treatment of hGCs-PTEN^KD^, the mRNA expression levels of NRF2, HO-1, PI3K and AKT were more substantially increased to 520%, 350%, 240% and 210%, respectively, over the levels observed in the siRNA control group. The protein level assay displayed the same results as the mRNA level analysis (Figure 8E). Furthermore, as shown in Figure 8F, hPMSCs and EGF inhibited the levels of ROS to 1.0% and 1.2%, respectively, after coculture with hGCs-PTEN^KD^, which were levels that were lower than those in the wild-type group (49%), as determined by FACS analysis.

## DISCUSSION

The lifespan of the ovaries controls female fertility, and that lifespan depends mainly on the size of the oocyte reserve [20]. Premature ovarian aging is one of the earliest negative physiological functions that occurs in ovaries; it is marked by an age-dependent decline in the quantity and quality of oocytes and a reduction in ovarian follicle reserve [21]. One study reported that in addition to the reduced oocyte pool, the decreases in oocyte quality and embryonic development potential also result in a decline in fertility in mice with premature age-related factors [22]. Human oocytes contain a large number of mitochondria, and a recent study found that substantially lower levels of mtDNA content induced ovarian insufficiency [23]. Moreover, increasing evidence supports the idea that oxidative stress may be a significant risk factor for reproductive disorders, and the progression of POI is an outcome of ROS overproduction [24]. Oxidative stress could cause ovarian aging, resulting from a decrease in antioxidants in the ovary in an age-related manner and the gradual accumulation of ROS [25]. The illumination of the mechanisms that underlie the reduction of oxidative stress and ovarian aging may play a vital role in potentially increasing the rate of laying performance and prolonging the ovarian lifespan.

Hormone replacement therapy (HRT) has been put into use for the treatment of patients with POI [7]. In addition to noticeable side effects, long-term application of HRT may contribute to a high risk of gynecological tumors. Recently, a substantial amount of research has been dedicated to studying perinatal stem cells for repair after injury, such as human amniotic fluid stem cells, human amniotic mesenchymal stem cells and human umbilical cord mesenchymal stem cells [11]. Moreover, human placenta mesenchymal stem cells (hPMSCs) have frequently been used in studies due to the following characteristics: easily obtained, abundant source material, low immunogenicity, easy culture, no oncogenicity and ethical restrictions, and convenient induction and expansion. However, the role of hPMSCs in the treatment of POI is poorly known. Our study showed that in a POI mouse model, the follicle number was nearly restored to normal after hPMSC transplantation at four stages (primordial, primary, secondary and antral follicle stage). Furthermore, we explored whether the serum indexes from the POI mouse model recovered to regular levels (Figure 1). To fill the bench-to-bedside gap, POI hGCs derived from patients and hPMSCs were cocultured to evaluate the preclinical efficacy. Our findings revealed that hPMSCs increased the marker expression of hGCs (FOXL2/CYP19A1 were related to ovary formation, and AMH/FSHR were related to follicular growth and maturation) (Figure 2). Our findings are the strong evidence of POI disease treatment by hPMSCs.

In our study, the results of the antibody microarray indicated that the growth factor EGF was released by hPMSCs at a high level (Figure 3B). EGF was highly expressed in the POI mouse ovaries after hPMSC transplantation (Figure 3C). To further address whether hPMSCs secreted EGF to positively affect POI, FACS analysis was used. The results indicated that EGF and hPMSCs reduced apoptosis and improved proliferation in POI hGCs (Figure 4). EGF and hPMSCs restored the levels of oxidative enzymes, antioxidant enzymes and ROS to normal levels in POI hGCs (Figure 5). Our data reinforce the work of another study that has shown that in vitro oocyte maturation and cumulus expansion can be induced by EGF and EGFR ligands [26]. Moreover, a previous study identified that native oocyte-secreted factors from less developed oocytes were less effective at inducing EGF responsiveness [27].

However, the regulatory mechanism of reduced ROS levels as a result of hPMSCs is currently unknown. NRF2 is a key protective component for oxidative stress resistance [28], and HO-1 is highly upregulated by numerous stimuli, such as nitric oxide cytokines, modified lipids, and other factors [28]. Our studies revealed that the expression levels of NRF2 and HO-1 were more dramatically increased by hPMSCs and growth factors (EGF) in vitro than they were in vivo (Figures 6 and 7). In addition, our results revealed a substantial increase in the expression levels of PI3K/AKT and a decrease in the expression level of PTEN induced by hPMSCs and EGF (Figure 6B and 7B). These results may explain the regulation of proliferation and apoptosis by hPMSCs and EGF. Furthermore, hPMSCs and EGF only partially reduced the ROS level in the hGCs-NRF2^KD^ group. Nevertheless, hPMSCs and EGF clearly inhibited the ROS level in the hGCs-PTEN^KD^ group (Figure 8). The results demonstrated that the effect of ROS in POI disease was resisted by EGF through the NRF2/HO-1 pathway. Several lines of evidence support our results and suggest that NRF2/HO-1 deficiency leads to DNA repair and aging, apoptosis, and embryonic death [29]. Our results showed that abnormal HO-1 expression could induce ovulation failure and infertility [30]. Previous research has also supported our results that maintaining optimal levels of NRF2 activity has a crucial role in proper cell function, because it deals with oxidative and reductive stress to maintain redox homeostasis [31].

In summary, this is the first study to explore the relationship between hPMSCs and POI. Mechanistic insights show how hPMSCs positively impact POI via secreting EGF. Our present study demonstrated that EFG derived from hPMSCs inhibited ROS by upregulating NRF2 and HO-1 expression and by inhibiting PTEN expression. Therefore, our study suggests that ovarian function in POI is improved by hPMSCs releasing EGF and that ROS are inhibited by growth factors through activation of the NRF2/HO-1 signaling pathway (Supplementary Figure 2).

## MATERIALS AND METHODS

### Collection and culture of hPMSCs

Individuals who had delivered by elective cesarean section at week 38 of gestation were included for the supply of human placentas. All participants were healthy Chinese women ranging in age from 25 to 35. The exclusion criteria were as follows: a history of infection, obstetric complications or underlying diseases (including those associated with syphilis, human immunodeficiency virus, hepatitis B virus, hepatitis C virus, hypertension, pregnancy-induced hypertension diabetes, placenta previa, gestational diabetes, or threatened premature delivery). The study protocol was approved by the Ethics Committee for Clinical Research at the Suzhou Hospital affiliated with Nanjing Medical University. We obtained informed consent from all study participants. After being collected and immediately placed in solution, human placental samples were extensively washed in PBS containing antibiotic-antimycotic (100 mg/ml streptomycin and 100 U/ml penicillin G; Thermo Fisher Scientific, Waltham, MA) for one hour on ice. After removal from the amniotic membrane and the placenta was divided into quadrants, and the chorionic plate and villous chorion were minced to less than 1 mm. To release cells, trypsin was used to enzymatically digest the minced sample (ten grams) in for 60 min at 37 °C culture medium that contained 4 mg/ml dispase (Thermo Fisher Scientific) and 3 mg/mL Collagenase Type IV (Thermo Fisher Scientific). The reaction was terminated after centrifugation at 600 g at room temperature for 5 min in medium containing 10% fetal bovine serum (FBS). Approximately 3×10^7^ cells were transferred into every 10 cm cell culture dish. Cells began to grow in an adherent fashion after 3 days, and cell clusters formed within one week. The 3^rd^-4^th^ passages of hPMSCs were used to perform all experiments.

### hPMSC phenotypic identification

To stain hPMSC-specific surface antigens, the following PE-conjugated antibodies were used: anti-human CD34, anti-human CD105, anti-human CD29, anti-human CD90 and anti-human CD73, as well as the appropriate corresponding isotype controls. These antibodies were purchased from BD, USA. Subsequently, we used FACS to analyze the stained cells, and the detailed description is identical to that of the methods section for FACS analysis. Differentiation kits for culture (Thermo, USA) were used to determine the pluripotency of hPMSCs.

### Collection of primary human ovarian granulosa cells (hGCs) from POI patients

As in our previous study [6], for the selection of the control group, normal patients less than 35-years-old with tubal occlusion were recruited. The following patients with POI who met the following inclusion criteria were used: women younger than 35 years with antral AMH < 1.1 ng/ml or FSH ≥ 10 mIU/ml and a follicle count < 5. Women with adverse factors were included, such as known abnormal karyotypes, ovarian surgery or previous autoimmune diseases. Informed consent and approval were obtained, and nhGCs were taken from patients with tubal occlusion and POI patients. All patients were treated with the GnRH antagonist Ganirelix (Merck Frosst, Canada) and recombinant FSH (Puregon, USA). Follicular development was monitored by vaginal ultrasound examination. We cultured hGCs as previously described [6].

### Development of a POI mouse model

Female ICR mice at eight weeks of age were purchased from Nanjing Medical University, and studies with them were approved by the Institutional Animal Care and Use Committee according to institutional guidelines. In accordance with our previous method, a POI mouse model was induced by employing a cyclophosphamide (CTX, 120 mg/kg, treatment two weeks) treatment method [6]. The animals were divided into four groups with 10 animals per group as follows: the wild-type group, the group treated with CTX and PBS, the group injected with hPMSCs, or the group injected with hPMSCs-CM. The hPMSCs (1 × 10^6^) were suspended in 100 μl of PBS and were transplanted into the mouse by the caudal vein. Conditioned medium (CM) from hPMSCs was injected into the mouse according to the same pattern, and then the follicle numbers and hormone levels were estimated. The experimental details are listed in the ELISA section of the Methods. The feeding conditions for the mice were the same as those described in our previous report [6].

### Coculture of hGCs with EGF and injection of these cells into POI mouse ovaries

As previously described, we grouped a portion of hGCs with different treatment concentrations, including the PBS culture control group and the EGF culture group (10 ng/ml, 20 ng/ml, or 40 ng/ml, R&D Systems). The hGCs were cultured with PBS or growth factors for 7 days. The POI mice were categorized into a PBS-injected control group and three concentration EGF-injected groups (0.1 μg/ml, 0.5 μg/ml, and 1.0 μg/ml, R&D system). The mouse ovary was injected with EGF or PBS, and the mice were sacrificed after 4 weeks to count follicles at different stages after performing hematoxylin and eosin staining.

### Assessment of ovarian function by comparing ovarian follicle counts

The mice were sacrificed at 0 to 4 weeks after cell transplantation. Bilateral ovaries were fixed in 10% formalin and then were embedded in paraffin. The 5-mm-thick sections were treated with hematoxylin and eosin (HE) staining. Follicles were detected and classified into four stages: primordial, primary, secondary and antral follicles. The calculation and comparison of the proportion of follicles from bilateral ovaries were performed among four groups with 10 follicles per group. We selected three representative sections from each ovary. To avoid counting a follicle twice, we only counted the follicles containing an oocyte. replicates performed, and the results are displayed as the fold change ± SD.

### Immunofluorescence staining

To assess the features of ovarian tissues, the following primary antibodies were used: anti-human HGF, anti-human BrdU, anti-human EGF, anti-human VEGF and anti-human bFGF. The above antibodies were purchased from Abcam, USA. After fixation in 4% paraformaldehyde (Sigma, USA) for 10 min at room temperature, ovarian sections were rinsed three times with PBS for 5 min each, and then they were subsequently permeabilized on ice for 10 min with 0.1% Triton X-100 (Sigma, USA). The sections were blocked at room temperature with 4% fresh BSA (Sigma, USA) in PBS for 30 min. Then, the cells were washed three times. The cells were incubated with primary antibodies overnight at 4 °C. Then, the cells were washed in PBS for 5 min before being incubated in the dark for 30 min with secondary antibodies (Cy2 or FITC, Jackson ImmunoResearch, West Grove). Then, the cells were analyzed using a fluorescence microscope (Olympus, Japan).

### Antibody microarray analysis

For cytokine estimation, we used a protein antibody array methodology with different antibodies (Human Growth Factor Array G1, RayBio Human Cytokine Antibody Array, RayBiotech, Inc., Norgross, GA); this enabled the determination of the expression profile of proteins in hPMSC-conditioned media (CM). The manufacturer’s instructions were followed by using one hundred micrograms of CM.

### FACS analysis

hPMSCs and hGCs were individually treated with trypsin-EDTA for 3 min to produce single cells. After fixation, according to the manufacturer’s instructions, hPMSCs and hGCs were processed with a Cytofix/Cytoperm Fixation/Permeabilization Solution kit (BD, USA). hPMSC staining was performed using PE-conjugated anti-human CD34, anti-human CD105, anti-human CD29, anti-human CD90 and anti-human-CD73 antibodies or their corresponding isotype control at 4 °C for 30 min. We purchased these antibodies from BD, USA. PE- or FITC-conjugated antibodies were used for hGC staining as follows: anti-human FOXL2, anti-human FSHR, and anti-human AMH (acquired from Thermo, USA), anti-human Ki67, anti-human ROS and anti-human Annexin V antibodies (purchased from Abcam, USA), as well as anti-human CYP19A1 (Abgent, USA) or their corresponding isotype control as previously mentioned [6]. A flow cytometer (Beckman, USA) was used to analyze these stained cells. The experiments had three replicates, with the results shown as fold change ± SD.

### Enzyme-linked immunosorbent assay (ELISA) analysis

Plasma from POI mice was used for the evaluation of the expression levels of AMH, E2 or FSH by an ELISA kit (MyBioSource, USA), and the detailed experimental process was described in our previous study [6].

### The activities of oxidoreductases and antioxidases

Homogenization of mouse ovaries was performed in Na_2_PO_4_KCl, and then the resulting homogenate were used to measure the activities of oxidoreductases (MDA and LDH) and antioxidases (SOD, GR, CAT and GPx). According to our previous reports [5], the activities of these enzymes were assayed via LSBio and Cayman Chemical Company.

### Western blot (WB) analysis

hGCs or ovarian cells were lysed with lysis buffer (Beyotime Biotechnology, China). Then, 20 μg of protein was extracted and loaded onto 10% or 20% gels and fractioned via SDS-PAGE (sodium dodecyl sulfate-polyacrylamide gel electrophoresis). Next, the separated proteins were electroblotted onto polyvinylidene difluoride (PVDF) membranes (Millipore, USA). The membranes were incubated with primary antibodies (FSHR, AMH, FOXL2, SURVIVIN, CYP19A1, BCL2, CASPASE9, CASPASE3, PTEN, PI3K, AKT, NRF2, p-AKT, HO-1, and GAPDH from Abcam) at 4 °C overnight, which was followed by incubation with secondary antibodies for 2 h at room temperature. The protein-level marker expression of each sample was detected with enhanced chemiluminescence (Pierce ECL Western blotting Substrate, Thermo Fisher, USA) and scanned by a chemiluminescence detection system (Tanon, China). ImageJ software (National Institutes of Health, USA) was used to analyze the signal intensity of the band of interest in the grayscale images. Experiments were repeated three times. The results are presented as the fold change ± SD.

### Gene silencing with RNA interference

For PTEN or NRF2 knockdown, hGCs were transfected with an siRNA (Thermo Fisher, USA) targeting human PTEN or NRF2 using Lipofectamine 2000 (Invitrogen, USA) according to the manufacturer’s instructions. A non-silencing scrambled siRNA was used to establish a negative control. WB analysis was performed to verify the knockdown efficiency by detecting target protein levels.

### Statistical analysis

One-way ANOVA was performed using SPSS 17.0 software. The means ± SD were used to show results, and *p* < 0.05 was regarded as significantly different.

## Supplementary Material

Supplementary Figures

Supplementary Table 1
