# Supplementary material for: EGF released from human placental mesenchymal stem cells improves premature ovarian insufficiency via NRF2/HO-1 activation
Source: Aging (Albany NY). 2020 Feb 10;12(3):2992–3009. doi: 10.18632/aging.102794 (PMC7041770; doi:10.18632/aging.102794)
Supplement: Supplementary Figures [file aging-12-102794-s002..pdf]

SUPPLEMENTARY FIGURES

Supplementary Figures

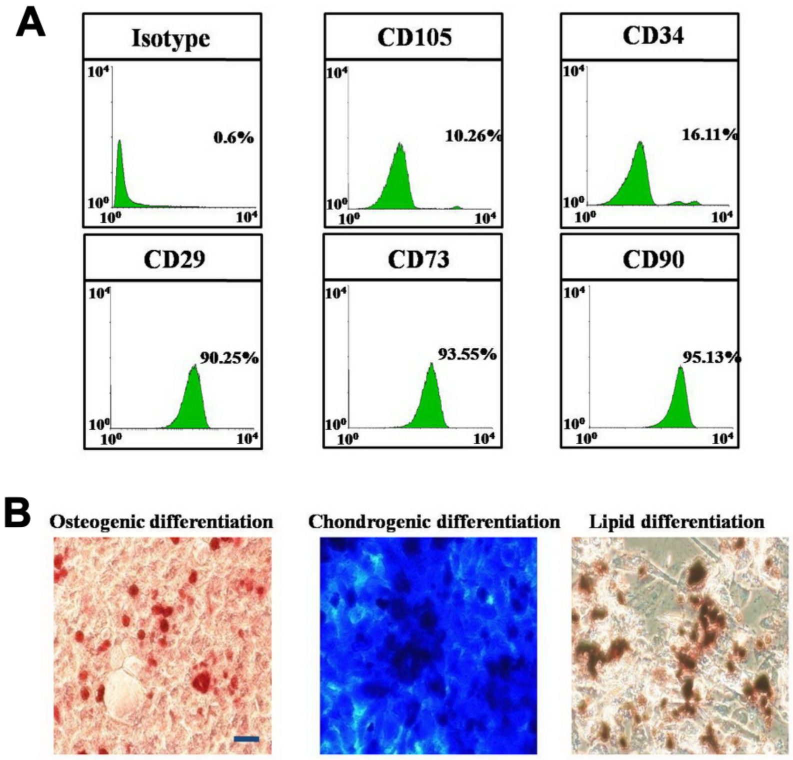

Supplementary Figure 1. Characterization of hPMSCs was tested. (**A**) The expression levels of CD105, CD29, CD34, CD73, and CD90 in hPMSCs were detected by flow cytometry. (**B**) hPMSCs can differentiate into adipocytes (oil red), osteoblasts (alizarin red) and chondroblasts (alcian blue) under standard in vitro differentiation conditions. Scale bars = 10 μm.

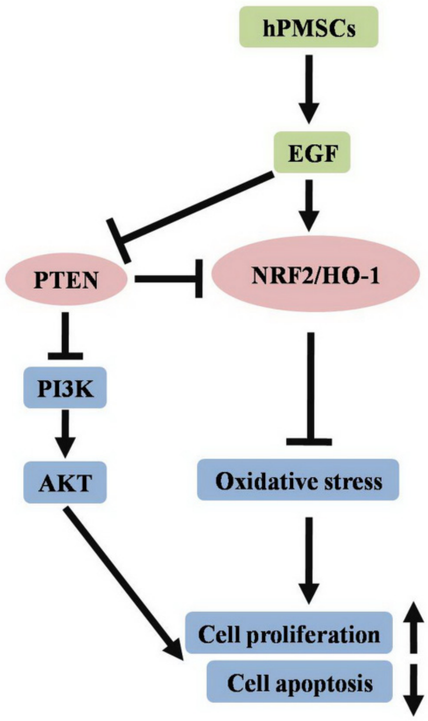

Supplementary Figure 2. Proposed model for the interaction between NRF2/HO-1 and POI disease. EGF derived from hPMSCs promoted the mRNA and protein expression of NRF2/HO-1 and inhibited the mRNA and protein expression of PTEN in POI.
