# Supplementary material for: EGF released from human placental mesenchymal stem cells improves premature ovarian insufficiency via NRF2/HO-1 activation
Source: Aging (Albany NY). 2020 Feb 10;12(3):2992–3009. doi: 10.18632/aging.102794 (PMC7041770; doi:10.18632/aging.102794)
Supplement: Supplementary Table 1 [file aging-12-102794-s001..pdf]

SUPPLEMENTARY TABLES

Supplementary Table 1. Designations, sequences, and the sizes of real-time PCR amplicons.

Name	Sequence from 5′-3′	Size (bp)
PI3K (H) Fw	TCCCTGACTTCTCCCCTTGT	167
PI3K (H) Rev	AGAGCTCCAAAGCAGCAGAG	
AKT (H) Fw	GGCCCGAAGGACGGGA	106
AKT (H) Rev	TGAGTTGTCACTGGGTGAGC	
PTEN (H) Fw	TTTTGAGGTGTTTGGGTTTTTGGT	124
PTEN (H) Rev	ACACAATCACATCCCAACACCA	
NRF2 (H) Fw	TCAGTCAGCGACGGAAAGAG	135
NRF2 (H) Rev	GTGGGCAACCTGTCTCTTCAT	
HO-1 (H) Fw	GCCTCACTGGCAGGAAATCA	120
HO-1 (H) Rev	AGGTAGCGGGTATATGCGTG	
GAPDH (H) Fw	GAAGGTCGGAGTCAACGGATTT	223
GAPDH (H) Rev	CTGGAAGATGGTGATGGGATTTC	
PI3K (M) Fw	TGGGAGAGAGCAGGCAAATTA	188
PI3K (M) Rev	ACACCCCAGCCAATCAAGTC	
AKT (M) Fw	TAGGCCCAGTCGCCCG	226
AKT (M) Rev	AGGTGCCATCGTTCTTGAGG	
PTEN (M) Fw	GTGGTGGAACTTGCAATCCT	240
PTEN (M) Rev	AGGTTTCCTCTGGTCCTGGT	
NRF2 (M) Fw	GGACATGGAGCAAGTTTGGC	165
NRF2 (M) Rev	TCCAGCGAGGAGATCGATGA	
HO-1 (M) Fw	GAAATCATCCCTTGCACGCC	122
HO-1 (M) Rev	CCTGAGAGGTCACCCAGGTA	
GAPDH (M) Fw	TTCCAGTATGACTCTACCCACGGCA	137
GAPDH (M) Rev	GCACCAGCATCACCCCATTTG	

H=Human; M=Mouse.
